# Supplementary material for: Comparative analysis of Egypt’s malaria elimination strategies and implementation science: Pathways to achieve malaria-free status for other African countries
Source: PLoS One. 2025 Jul 31;20(7):e0329550. doi: 10.1371/journal.pone.0329550 (PMC12312880; doi:10.1371/journal.pone.0329550)
Supplement: Attachment — Submitted filename: Review report PLOSE ONE.docx [file pone.0329550.s001.docx]

**Review report**

Re: Comparative analysis of Egypt’s malaria elimination strategies and implementation science: Pathways to achieve malaria-free status for other African countries

Elendu et al.

**General comments**:

*The authors aimed to provide an overview of how Egypt achieved malaria-free status. They emphasised the proactive use of larval source management, the evidence-based implementation of vector control, and the provision of antimalarial treatments in areas with high malaria burdens. Additionally, they recommended that other countries adopt Egypt's strategies to attain their malaria elimination goals, which is worthy.*

*It would have been more beneficial if the authors had discussed how the Egyptian health system was established, the sources of funding for malaria interventions, and how the country followed WHO policies and guidelines. Additionally, exploring the connection between economic development and societal changes related to malaria reduction and elimination and the relationship between malaria and poverty would have been helpful. Egypt has a relatively stable economy and predominantly lives in urban settings, where people's awareness is relatively better, and communication is easy.*

*Housing transformation, economic development, the advantage of the climate, mosquito vectorial potential, and the availability of water bodies are also critical.*

*The authors didn’t provide data on the composition of Plasmodium species and the vectorial capacity of the malaria vector in Egypt compared to the other African countries they mentioned. The mosquito and human behavioural dimensions should also be considered alongside the existing intervention.*

*How do Egypt's strategies for implementing interventions differ from those of other African countries regarding adhering to WHO policies and guidelines? The World Health Organization (WHO) is the governing body that establishes these protocols. For instance, countries are instructed to select interventions—indoor Residual Spraying (IRS) or Insecticide-Treated Nets (ITNs)—based on their malaria burden, reflecting WHO's focus on addressing financial constraints. Has Egypt had any experiences overcoming these challenges that could be shared with other nations?*

*The authors argue that ….* Unlike some sub-Saharan African countries where the predominant vectors, such as *Anopheles gambiae*, exhibit insecticide resistance, Egypt benefitted from focusing on localized and context-specific measures against *Anopheles sergenti* [17][18]. These measures included environmental modifications, such as removing breeding sites and strategically deploying insecticide-treated nets (ITNs) and indoor residual spraying (IRS)….*What was unique here apart from the vector?*

*It would be nice if the authors could argue what other African countries can do to eliminate malaria using existing interventions, as Egypt did with the consideration of the financial constraints and low in-house funding of several African countries, including those mentioned as examples. How do the authors compare the population at risk, the malaria endemicity, and the suitability of environments for vector breeding in Egypt to other African countries?*
