# Supplementary material for: Comparative analysis of Egypt’s malaria elimination strategies and implementation science: Pathways to achieve malaria-free status for other African countries
Source: PLoS One. 2025 Jul 31;20(7):e0329550. doi: 10.1371/journal.pone.0329550 (PMC12312880; doi:10.1371/journal.pone.0329550)
Supplement: Attachment — Submitted filename: Reviewers.docx [file pone.0329550.s003.docx]

We sincerely thank Reviewer #1 for their thoughtful and detailed feedback, which has significantly strengthened our manuscript. In response to your comments, we expanded the discussion of how strategies such as vector control, antimalarial treatment, and surveillance were implemented at the grassroots level. Specifically, we provided more detailed descriptions of community engagement efforts, the role of community health volunteers, challenges faced by local healthcare providers such as staff shortages and supply chain disruptions, and the contextual strategies used to build trust and sustain participation.

We also deepened the discussion of Egypt’s political leadership, highlighting not only the government's commitment but also specific leadership practices, mechanisms for inter-sectoral coordination, and funding strategies that supported the malaria elimination efforts.

Comparative analysis was strengthened by adding a brief comparison between Egypt’s approach and those of other African countries like Nigeria and Mozambique. This comparison draws on intervention efficacy, cost-effectiveness, and contextual differences in healthcare system strength, illustrating how Egypt’s experience may or may not be generalizable to countries with more severe endemic burdens.

In response to the your suggestion regarding critical reflection on the broader applicability of Egypt’s strategies, we have added a discussion about the limitations of applying Egypt’s model to other African contexts, considering variations in endemicity, healthcare infrastructure, and socioeconomic factors.

We expanded our treatment of climate change and resistance issues by providing concrete examples of Egypt’s proactive measures, such as ongoing insecticide resistance monitoring and shifts to alternative insecticides and treatment regimens. We cited recent relevant literature to support these updates, specifically:

Abdul-Rahman, Toufik, et al. "Improving diagnostics and surveillance of malaria among displaced people in Africa." International Journal for Equity in Health 24, no. 1 (2025): 22.

Ali, Tehreem, et al. "The fight against malaria: a new hope with the R21 vaccine." IJS Global Health 7, no. 2 (2024): e0423.

Finally, regarding sustainability, we expanded the discussion on Egypt’s long-term mechanisms to maintain malaria-free status. We included detailed explanations of the country’s surveillance strategies, investments in continued public health education, integration of malaria monitoring into primary healthcare services, and research initiatives focused on early detection of drug and insecticide resistance.

We believe these revisions have substantially strengthened the manuscript and aligned it closely with the your valuable recommendations.

We sincerely thank Reviewer #2 for their detailed and constructive feedback, which has been immensely helpful in improving the clarity, structure, and scientific rigor of our manuscript. In response to your comments, we have made substantial revisions throughout the paper. We have now explicitly used an organizing framework, incorporating the RE-AIM model to better link context, strategies, and outcomes, and to ensure a more systematic presentation of our findings.

In the Abstract, we have added clear information on the implementation outcomes evaluated, such as fidelity, feasibility, and adaptability, and we have articulated the specific implementation strategies that led to these outcomes. The Background section has been reorganized to focus on the initial malaria problem in Egypt, the country’s final outcome, and the rationale for using an implementation science approach. We have moved the description of Egypt’s strategies from the Background to the Results section, as suggested. Additionally, we have shortened and clarified the statement of concrete aims to clearly outline the implementation outcomes being measured, and we clarified whether we focused solely on implementation strategies or included contextual factors.

In the Methods section, we have reorganized the content to strictly describe how the study was planned and conducted. We now clearly provide the study location, describe the evidence-based interventions (EBIs), explain how quantitative and qualitative data were obtained, and detail participant recruitment, data collection tools, and analysis procedures for each data source. We clarified the eligibility criteria and explained what was meant by “relevance to the implementation science framework.” Moreover, we now detail how each implementation outcome (fidelity, feasibility, adaptability, and acceptability) was measured, specifying whether preexisting validated tools were used or new instruments were developed. To enhance clarity, we also organized the implementation outcomes and corresponding measures into a new table. Sentences that previously belonged in the Discussion have been moved appropriately.

The Results section has been substantially reorganized for improved clarity and flow, following the chosen framework. We clarified the participant groups for evaluating implementation versus intervention effectiveness, defined key terms such as “access” and “adherence,” and provided detailed explanations for the categories reported in the tables (e.g., socioeconomic status). We also corrected the description of mechanisms versus strategies and clarified how fidelity was measured. Indicators were now properly aligned with their corresponding outcomes to avoid confusion.

In the Discussion section, we revised the content to reflect all the above changes, providing a more detailed and coherent interpretation of our findings, properly structured and guided by the organizing framework.

Once again, we deeply appreciate Reviewer #2’s feedback, which led to significant improvements in the manuscript.

We sincerely thank Reviewer #3 for their thoughtful and constructive feedback, which helped us to critically refine and broaden the scope of our manuscript. In response, we have incorporated additional discussion points that more comprehensively explore the establishment of Egypt’s health system, sources of funding for malaria interventions, and the extent to which Egypt followed WHO policies and guidelines. We have added a new subsection discussing the economic development of Egypt, societal changes, and the connection between malaria reduction and poverty alleviation. Furthermore, we now highlight factors such as housing transformation, climatic advantages, mosquito vectorial potential, and the availability of water bodies, integrating these into our analysis of Egypt’s successful malaria elimination strategy.

We also expanded our discussion to include the composition of Plasmodium species and the vectorial capacity of the malaria vectors in Egypt compared to those in other African countries. Specifically, we elaborated on how human and mosquito behavioral dimensions were addressed in Egypt and their relevance to intervention outcomes.

In addressing the comment on how Egypt's strategies differed from those of other African countries, we have now detailed how Egypt’s adherence to WHO policies was adapted in a proactive and context-specific manner, compared to the more rigid or resource-limited adaptations in other African settings. We discussed how Egypt managed financial constraints, leveraged local resources, and strategically implemented WHO-recommended interventions, such as IRS and ITNs, based on local epidemiological data rather than purely national-level assumptions.

We clarified the unique aspects of Egypt’s approach beyond vector type, noting how strategic environmental modifications, early surveillance, community mobilization, and integrated vector management were effectively tailored to the Anopheles sergenti ecology.

Finally, we strengthened our argument regarding what other African countries can do to eliminate malaria using existing interventions, despite financial and infrastructure challenges. We now include a comparative analysis of population at risk, malaria endemicity, and the ecological suitability for vector breeding between Egypt and other African countries, thus providing a more nuanced understanding of the transferability of Egypt’s strategies.

We sincerely appreciate Reviewer #3’s valuable insights, which helped us make the paper more comprehensive, comparative, and practical for informing malaria elimination efforts across Africa.
