# Supplementary material for: Comparative analysis of Egypt’s malaria elimination strategies and implementation science: Pathways to achieve malaria-free status for other African countries
Source: PLoS One. 2025 Jul 31;20(7):e0329550. doi: 10.1371/journal.pone.0329550 (PMC12312880; doi:10.1371/journal.pone.0329550)
Supplement: Attachment — Submitted filename: Reviewers_auresp_2.docx [file pone.0329550.s004.docx]

Reviewer #1: Thank you very much for your positive feedback and kind words. We sincerely appreciate your thoughtful review and are glad that the revisions addressed your comments and concerns. Your support and encouragement mean a great deal to us.

Reviewer 2#: Thank you for your constructive feedback and for acknowledging the improvements made to the manuscript. We appreciate your continued engagement, which has helped strengthen the quality and clarity of our work.

Regarding the use of RE-AIM and Proctor’s frameworks, we have revised the Methods section to clarify that a hybrid framework approach was employed. Specifically, RE-AIM served as the overarching structure for evaluating the public health impact of Egypt’s malaria elimination program, while Proctor’s implementation outcomes framework informed the operational definitions and measurement of specific implementation outcomes. We also updated the title of Table 1 to reflect this integrated approach.

To address the comment on data availability, we revised the Data Availability Statement to clarify that all data supporting the study’s findings were obtained from publicly available sources, including program reports and published literature, and that no primary data were collected. This addresses the presence of qualitative data while affirming that no new or participant-derived data were generated.

We have also clarified in the manuscript that all data were sourced from public domain materials, such as WHO and national malaria program reports. Although the study focuses on Egypt’s malaria program, the data were obtained from secondary sources, and the ethical approval from the Nigerian Research Ethics Committee was sought because the authors are affiliated with Nigerian institutions and the qualitative analysis did not involve direct interaction with human subjects.

Finally, we have shortened the Future Directions section to improve brevity and balance the overall manuscript length in line with your recommendation.

We are grateful for your valuable insights and hope the revised manuscript now meets your expectations.
